# A Fast Data Transmission Method of Vehicle-Road Cooperative Communication Based on the Clustering Algorithm

**DOI:** 10.1155/2022/9162360

**Published:** 2022-09-01

**Authors:** Weifeng Wang, Zijian Wang, Yang Xu, Xinpeng Yao, Yulin Ma

**Affiliations:** ^1^College of Civil and Transportation Engineering, Hohai University, Nanjing 210098, China; ^2^Shandong Key Laboratory of Smart Transportation (Preparation), Jinan 250101, China; ^3^School of Engineering, Ocean University of China, Tsingtao 266100, China; ^4^School of Mechanical Engineering, Anhui Polytechnic University, Wuhu 241000, China

## Abstract

In order to improve the security and stability of data transmission in vehicle-road cooperative communication and reduce the error rate of data transmission, a fast data transmission method based on the clustering algorithm is proposed. First, the multisensor information acquisition method of the vehicle networking is used to realize data acquisition and data structure analysis of the vehicle-road cooperative communication, and a data transmission channel model for vehicle-road cooperative communication is constructed. The interference suppression in the data transmission process of the vehicle-road cooperative communication is realized by the matched filter detection algorithm. Then, according to the symbol characteristic distribution of the vehicle-road cooperative communication channel, the baud interval equalization method is used to realize the piecewise equalization adjustment of data transmission for the vehicle-road cooperative communication. With the adaptive error compensation and channel fading suppression, the K-means clustering algorithm is used to carry out the coordinated adjustment to data packets during the rapid data transmission of the vehicle-road cooperative communication. Finally, the adaptive control of data transmission for the vehicle-road cooperative communication is carried out according to the dynamic distribution characteristics of the clustering center so as to reduce the influence of channel fading and intersymbol interference caused by channel spread. The simulation results show that the bit error rate of this method is kept at about 0.05, and the data transmission rate continues to increase, most of which remain above 0.95. This method has strong anti-interference ability for the rapid data transmission of vehicle-road cooperative communication, with lower communication bit error rate, less end-to-end time delay, and higher stability and accuracy of data transmission.

## 1. Introduction

With the development of vehicle networking technology and vehicular ad hoc network (VANET), people attach great importance to the research on the security and stability of data transmission in vehicle-road collaborative communication. With the channel balance control of vehicle-road collaborative communication, a data transmission method with high stability and strong anti-interference is adopted to realize the optimal control of vehicle-road collaborative communication and improve the collaborative control ability for the vehicle-road collaborative communication in data transmission. It is of great significance to study the rapid data transmission method of vehicle-road cooperative communication in the design of obile ad hoc network (MANET) for vehicle-road cooperative control and vehicle networking [1].

The data transmission of vehicle-road cooperative communication has the characteristics of nonstationarity and multipath so that the fast data transmission of vehicle-road cooperative communication is affected by intersymbol interference and multipath, and the output stability is not good. Thus, it is necessary to design a data transmission control model for vehicle-road cooperative communication to improve the stability of fast data transmission of the vehicle-road cooperative communication with channel equalization adjustment. At present, the fast data transmission methods of vehicle-road cooperative communication mainly include the data transmission control method of vehicle-road cooperative communication based on orthogonal frequency division multiplexing, the BPSK modulation method, the PSK modulation method, and so on [2–4]. Through time-frequency characteristic analysis and equalization control, the data transmission control of vehicle-road cooperative communication is realized. Reference [5] has proposed a method of vehicle-road cooperative communication and interference suppression based on the PTS-clipping algorithm. In the method, the piecewise fitting control method is used to design the channel equalization and suppress the intersymbol interference in the process of data transmission of vehicle-road cooperative communication, which improves the output signal-to-noise ratio and reduces the intersymbol interference, but the stability and real-time performance of this method are not good. Reference [6] has proposed a vehicle-road cooperative communication control method for the direct sequence spread spectrum. In this method, the direct sequence spread spectrum transmission method is used to transmit the data of vehicle-road cooperative communication in the spatial multipath channel, which reduces the output bit error rate, but the computational load is high. Reference [6] has proposed a vehicle-road cooperative communication control method based on matched filter detection. In this method, the interference suppression and filtering during data transmission of vehicle-road cooperative communication are analyzed by a matched filter detector, which improves the stability and real-time performance of data transmission of vehicle-road cooperative communication, together with error compensation, but the packet reception performance of this method is not good under the background of strong interference.

In view of the above problems, a fast data transmission method of vehicle-road cooperative communication based on clustering algorithm is proposed in this study. First, the multisensor information collection method of vehicle network is used to realize data acquisition and data structure analysis of vehicle-road cooperative communication. Then according to the symbol characteristic distribution of the vehicle-road cooperative communication channel, the baud interval equalization method is used to realize the piecewise equalization adjustment to data transmission for the vehicle-road cooperative communication. With the adaptive error compensation and channel fading suppression, the K-means clustering algorithm is used to carry out the coordinated adjustment of data packets during the rapid data transmission of the vehicle-road cooperative communication, and the Matlab simulation analysis method is used to realize the fast data transmission of vehicle-road cooperative communication. The results demonstrate the superior performance of the proposed method in improving the control ability of vehicle-road cooperative communication.

## 2. Channel Model of Vehicle-Road Cooperative Communication and Signal Analysis

### 2.1. Channel Model

**Figure 1:**
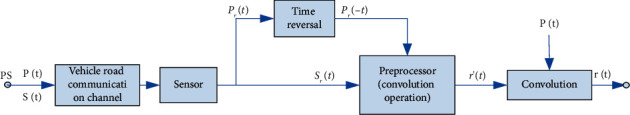
Structure model of data input and output of vehicle-road cooperative communication.

In order to realize fast data transmission and optimal control of vehicle-road cooperative communication based on the clustering algorithm, a channel model of vehicle-road cooperative communication is constructed based on routing protocol control and channel model equalization design of vehicle-road cooperative communication network. The single copy routing algorithm SimBet is used to reorganize the data transmission channel model of vehicle-road cooperative communication. With the passive time reversal mirror (PTRM) and BPSK modulation technology, the channel model of vehicle-road cooperative communication is established [6]. According to the attenuation of the data transmission bandwidth of the communication network, the weighted vector of the channel and the frequency component of the output signal are obtained. Through the convolution operation, the communication data transmission and equalization design are realized. The structure model of data input and output is shown in Figure 1.

According to the structure model of vehicle-road cooperative communication data input and output shown in Figure 1, the optimal distribution control of vehicle-road cooperative communication data time series is carried out based on the attenuation of communication network data transmission bandwidth. The vehicle network multisensor information collection method is used to realize the data acquisition and data structure analysis of vehicle-road cooperative communication. Assuming that the vehicle-road cooperative communication data signal is *p*(*t*) and the cooperative communication data sequence of PS waiting for a period of time *Tg* to be transmitted is *S*(*t*), the output processed by the reversal mirror is as follows after Wigner–Ville distribution detection and time reversal:

(1) $$\begin{array}{c} p_{ri}\left(t\right)=p\left(t\right)*h_{i}\left(t\right)+n_{pi}\left(t\right), \end{array}$$

where *h*_*i*_(*t*) represents the attenuation coefficient of impulse response characteristic quantity *p*(*t*) of vehicle-road cooperative communication data in the multipath channel. The channel equalization control of vehicle-road cooperative communication data transmission is carried out on a continuous sliding window to obtain the symbol sequence of vehicle-road cooperative communication data *S*_*r*_(*t*) after matched filtering:

(2) $$\begin{array}{c} S_{r}\left(t\right)=S\left(t\right)*h\left(t\right)+n_{s}\left(t\right), \end{array}$$

where *n*_*s*_(*t*) represents the interference noise on the data collection bandwidth of the vehicle-road collaborative communication, and the relevant matched filter detection output is obtained through a preprocessor *P*_*r*_(−*t*):

(3) $$\begin{array}{c} S_{ri}\left(t\right)=S\left(t\right)*h^{\prime }{}_{i}\left(t\right)+n_{si}\left(t\right), \end{array}$$

where *h*′_*i*_(*t*) is the response function after filtering in *S*(*t*), and the bandwidth of the data transmission array element sequence of vehicle-road cooperative communication is

(4) ri′t=Stri∗p−tri =St∗p−t∗h′it∗hi−t+nt1i,

where *S*(*t*) is the local signal, *S*_*ri*_(*t*) is the multipath signal, *p*_*ri*_(−*t*) is power spectral density, and *n*_1*i*_(*t*) is the noise component. The signal component in the extended signal *r*(*t*) of the vehicle-road cooperative communication in the spatial multipath channel is approximately the information signal waveform *S*(*t*), and the output interference suppression component is

(5) $$\begin{array}{c} n_{1i}\left(t\right)=S\left(t\right)*h^{\prime }{}_{i}\left(t\right)*n_{pi}\left(-t\right)\\ +n_{si}\left(t\right)*p\left(-t\right)*h_{i}\left(-t\right)\\ +n_{si}\left(t\right)*n_{pi}\left(-t\right), \end{array}$$

where *n*_*pi*_(−*t*) represents the noise component on the *p*-th symbol sequence, and *n*_*si*_(*t*) represents the noise component on the *s* symbol sequences. According to the above processing, the data transmission channel model of vehicle-road cooperative communication is constructed, and the anti-interference design of signal transmission is realized by the matched filter detection algorithm.

### 2.2. Transmission Signal Analysis

In order to obtain the data transmission gain of vehicle-road cooperative communication, the subsection equalization adjustment of vehicle-road cooperative communication data transmission is realized. It is assumed that the vehicle-road cooperative communication data are transmitted to each node *b*_*i*_, and the number of pulse frames for the impulse response of the channel is *N*_*f*_, then the time required *T*_spray_=max(*t*_*i*_) for the diffusion stage is obtained by the auto-correlation matching filter detection. The extended signal of vehicle-road cooperative communication data output is

(6) $$\begin{array}{c} r\left(t\right)=\sum_{i=1}^{M}r_{i}{}^{\prime }\left(t\right)*p\left(t\right)\\ \;=S\left(t\right)*p\left(t\right)*p\left(-t\right)*\sum_{i=1}^{M}h^{\prime }{}_{i}\left(t\right)*h_{i}\left(-t\right)+\sum_{i=1}^{M}n_{i}\left(t\right), \end{array}$$

where *n*_*i*_(*t*) is also the noise interference term of the data transmission channel for vehicle-road cooperative communication, *n*_*i*_(*t*)=*n*_1*i*_(*t*)*∗p*(*t*). The forwarding delay depends on the diffusion delay. Therefore, in the data transmission process of vehicle-road cooperative communication, the fuzzy function is introduced and the feature clustering algorithm is used. The clustering algorithm is a classic algorithm to solve the clustering problem, which is simple and fast. For dealing with large data sets, the algorithm maintains scalability and efficiency. When the cluster is close to Gaussian distribution, its effect is better. The fuzzy matching function of data transmission of vehicle-road cooperative communication is obtained as

(7) $$\begin{array}{c} H\left(t\right)=\overset{\wedge }{h}\left(t\right)*p\left(t\right)*p\left(-t\right)\\ =\left(\sum_{i=1}^{M}h^{\prime }{}_{i}\left(t\right)*h_{i}\left(-t\right)\right)*p\left(t\right)*p\left(-t\right), \end{array}$$

where $\overset{\wedge }{h}\left(t\right)$ and *p*(*t*)*∗p*(−*t*) approximate the impulse response function *δ*(*t*); *p*(*t*) and *p*(−*t*) indicate the spectral components of the impulse response signal taken on the positive and negative sequences, respectively. *∗* is convolution operation. By the time-frequency analysis method and through calculation, *h*′_*i*_(*t*) is calculated through *h*_*i*_(*t*), and the signal components of the vehicle-road cooperative communication channel are processed by feature focusing, and the following result is obtained:

(8) $$\begin{array}{c} \left|s\left(f\right)\right|=A\sqrt{\frac{1}{2k}}\left\{{\left[c\left(v_{1}\right)+c\left(v_{2}\right)\right]}^{2}+{\left[s\left(v_{1}\right)+s\left(v_{2}\right)\right]}^{2}\right\}, \end{array}$$

where *A*(*t*) is the amplitude of the vehicle-road cooperative communication signal, *f*_0_ is the initial transmission frequency of the vehicle-road cooperative communication channel, *k*=*B*/*T* is the BPSK modulation component of the vehicle-road cooperative communication, and *B* is the frequency modulation signal bandwidth. The spread spectrum algorithm is adopted to obtain the information flow of the output signal *a*(*t*) in the delay time interval *T*_*a*_. Accordingly, the data clustering algorithm is adopted to obtain the data transmission gain of the vehicle-road cooperative communication as follows:

(9) $$\begin{array}{c} E={\left\|x\left(t\right)\right\|}^{2}={\sum_{j}\sum_{k}\left|C_{j}\left(k\right)\right|}^{2}=\sum_{j}E_{j}, \end{array}$$

where *x*(*t*) is the time series of data transmission signal of vehicle-road cooperative communication, *C*_*j*_(*k*) is the data delivery delay, and *E*_*j*_ is the amplitude modulation parameter. According to the above channel and signal analysis, the baud interval equalization method is adopted to realize the piecewise equalization adjustment for data transmission of vehicle-road cooperative communication according to the symbol characteristic distribution of the vehicle-road cooperative communication channel [7].

## 3. Optimization of Fast Data Transmission in Vehicle-Road Collaborative Communication

### 3.1. Data Transmission Channel Equalization of Vehicle-Road Collaborative Communication

The channel equalization model for vehicle-road cooperative communication is established by adaptive control of link forwarding protocol and baud interval equalization control [8]. In the process of forward and reverse driving, the time-frequency characteristic component of the vehicle-road cooperative communication signal received by the receiver is as follows:

(10) $$\begin{array}{c} s\left(t\right)=\text{cos}\left[2\pi f_{0}t+\pi \beta t^{2}+\psi_{0}\right], \end{array}$$

where *f*_0_ and *ψ*_0_ are the starting frequency and initial phase of the multipath channel of vehicle-road cooperative communication, respectively. The statistical characteristics of the units to be detected are estimated, and the spread spectrum allocation model of vehicle-road cooperative communication channel is constructed. The extended bandwidth loss is expressed as

(11) $$\begin{array}{c} X^{\prime }=\sum_{v=1}^{V}b_{v}X_{v}, \end{array}$$

where {*b*_*v*_, *v*=1,2, ⋯, *V*} is the impulse weighting coefficient of the data transmission channel of vehicle-road cooperative communication and *X*_*v*_ represents the distance ambiguity parameter. *p*(*t*) is introduced as the propagation attenuation coefficient. The multipath spectrum bandwidth of vehicle-road cooperative communication is *W*. By adopting the iterative learning and adaptive control methods, an equilibrium control mode for the multivehicle-road cooperative communication channel in multidimensional road network space is constructed [9], and the channel equilibrium adjustment output is as follows:

(12) $$\begin{array}{c} H\left(z\right)=\text{Am}\cdot \frac{1+2z^{-1}+z^{-2}}{\left(1-\rho e^{j\phi }z^{-1}\right)\left(1-\rho e^{-j\phi }z^{-1}\right)}, \end{array}$$

where *z* is the transfer function of vehicle-road cooperative communication, *A* is the amplitude of path loss, and *m* is the intersymbol interference. Using pole estimation, the signal output *x*(*n*) in the road network *ρe*^±*jϕ*^ is

(13) $$\begin{array}{c} x\left(n\right)=A\text{cos}\left(0.3\pi n+\varphi \right)+v\left(n\right), \end{array}$$

where *φ* is the output spread phase of vehicle-road cooperative communication and *v*(*n*) is the intersymbol interference caused by path spread. By introducing the multiscale characteristics of channel transmission, data transmission control and adaptive adjustment are carried out based on man-machine interaction control [10].

### 3.2. Data Clustering of Vehicle-Road Collaborative Communication and Transmission Optimization

**Figure 2:**
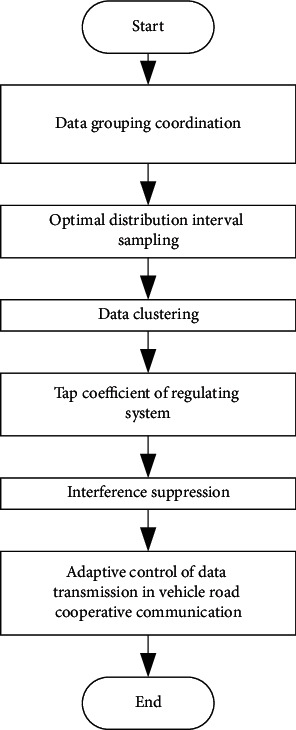
Flow chart.

Based on data transmission channel equalization of vehicle-road cooperative communication, data clustering is carried out to vehicle-road cooperative communication, and the specific flow chart is shown in Figure 2.

**Figure 3:**
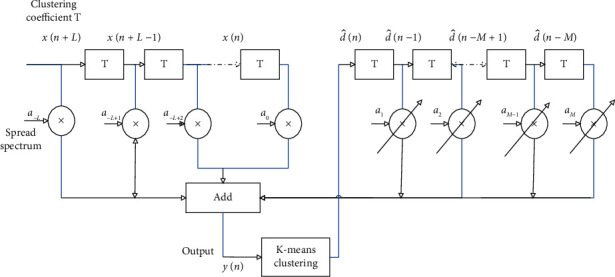
K-means clustering of data.

With the adaptive error compensation and channel fading suppression, the K-means clustering algorithm is used to carry out coordinated adjustment to data packets during the rapid data transmission of the vehicle-road cooperative communication. The K-means clustering algorithm is an iterative clustering analysis algorithm. Its step is to divide the data into *k* groups, randomly select *k* objects as the initial clustering center, then calculate the distance between each object and each subclustering center, and assign each object to the nearest clustering center. According to the attenuation of communication network data transmission bandwidth, the optimal distribution interval sampling of vehicle-road cooperative communication data time series is carried out [11], and the clustering function is obtained as follows:

(14) $$\begin{array}{c} s\left(t\right)=\sum_{i}b_{j}\sum_{j=0}^{N_{f}-1}p\left(t-iT_{s}-jT_{f}-c_{j}T_{c}\right), \end{array}$$

where *b*_*j*_ is the interference item of the vehicle-road cooperative communication data, *T*_*s*_ is the time sampling interval, and *c*_*j*_ is the transmission bandwidth of the vehicle-road cooperative communication data. Assuming that the similarity of the vehicle-road cooperative communication data is *h*(*n*), the nonlinear equilibrium parameter is *n*(*n*), the time domain loss is *y*(*n*), and the intersymbol interference is $\tilde{x}\left(n\right)$, then under the limited symbol rate, the channel aliasing and superposition are carried out by convolution *h*′_*i*_(*t*)*∗h*_*i*_(−*t*), and the data clustering is realized by the data K-means cluster shown in Figure 3.

With the clustering result of vehicle-road cooperative communication data in Figure 3 as the output, the spectral component of communication data output is as follows:

(15) $$\begin{array}{c} S\left(n_{j}\right)=\left(E_{\text{elec}}+E_{DF}\right)l\delta +E_{Tx\left(l,d_{j}\right)}\\ \;\;\;\;\;=\left(E_{\text{elec}}+E_{DF}\right)l\delta +lE_{\text{elec}}+l\varepsilon_{fs}d_{j}{}^{2}\\ \;\;\;\;\;=\left[\left(E_{\text{elec}}+E_{DF}\right)\delta +E_{\text{elec}}+\varepsilon_{fs}d_{j}{}^{2}\right]l, \end{array}$$

where *E*_elec_ is the superposition component of vehicle-road cooperative communication at the same time in the same phase, *E*_*DF*_ is the attenuation loss of multipath spread of vehicle-road cooperative communication, *E*_*Tx*(*l*, *d*_*j*_)_ is the channel impulse characteristic quantity of vehicle-road cooperative communication transmission, *ε*_*fs*_ is the frequency spectrum parameter, *l* is fuzzy information sequence, and *d*_*j*_ is the multipath component of detection signal. The tap coefficient of vehicle-road cooperative communication data transmission system is adjusted, and the blind equalization method is adopted to suppress interference. The adaptive control of data transmission in vehicle-road cooperative communication is carried out according to the dynamic distribution characteristics of clustering centers so as to reduce the effects of channel fading and intersymbol interference caused by the channel spread spectrum [12].

## 4. Simulation Experiment

**Figure 4:**
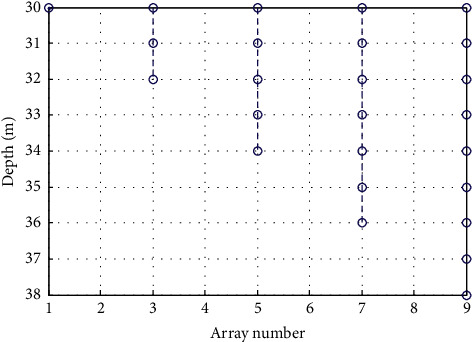
Distribution of transmission elements in vehicle-road cooperative communication.

In the experiment, the distance between vehicles in the motorcade was kept at 24–100 m, and the upper speed line *v*_max_ was 120 km/h. RFID was used as the sensor in the motorcade, and 100 receiving array elements were set to form the transmission array of vehicle-road cooperative communication. The independent variable *X* of data transmission in vehicle-road cooperative communication indicated the distance between two nodes in the link establishment time. The vehicle driving can be divided into two situations: forward driving and reverse driving. The communication range was [0,300), satisfying 0 ≤ *X* < 300. In vehicle-road cooperative communication, the symbol transmission rate was set at 1 k Baud. The maximum data transmission delay was set at 25 ms; the symbol distance was 100 m; the waiting time of vehicle-road cooperative communication data was *Tg*=100ms; the signal-noise ratio was fixed at 10 dB; the number of snapshots was changed from 200 to 2000. According to the above simulation environment and parameter settings, the data transmission optimization simulation analysis of vehicle-road cooperative communication was carried out, and the distribution of transmission array elements of vehicle-road cooperative communication is shown in Figure 4.

**Figure 5:**
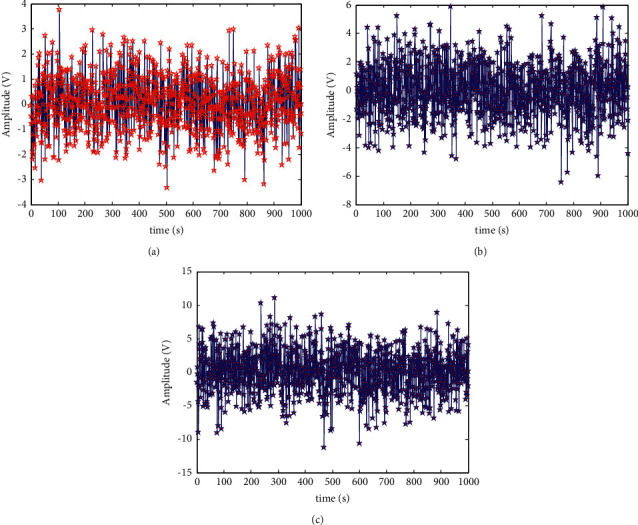
Output time series of vehicle-road cooperative communication data. (a) Transmission data sample 1 (SNR = 0 dB). (b) Transmission data sample 2 (SNR = −5 dB). (c) Transmission data sample 3 (SNR = −10 dB).

According to the array element distribution in Figure 4, the signal transmission model for vehicle-road cooperative communication was constructed, and the output time series of vehicle-road cooperative communication data was obtained by taking the input signal-to-noise ratio of −10 dB, −5 dB, and 0 dB, respectively, as shown in Figure 5.

**Figure 6:**
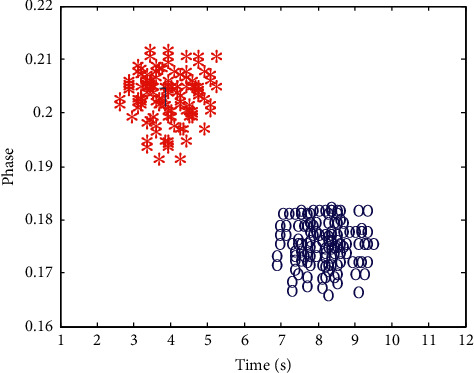
Data clustering results.

With the output time series of vehicle-road cooperative communication data shown in Figure 5 as a sample, the data were clustered. The clustering results are shown in Figure 6.

**Figure 7:**
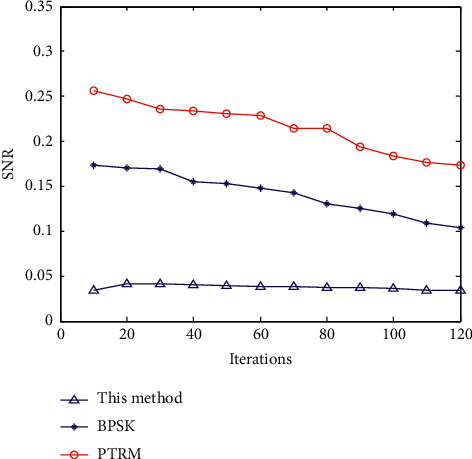
Bit error rate comparison.

**Figure 8:**
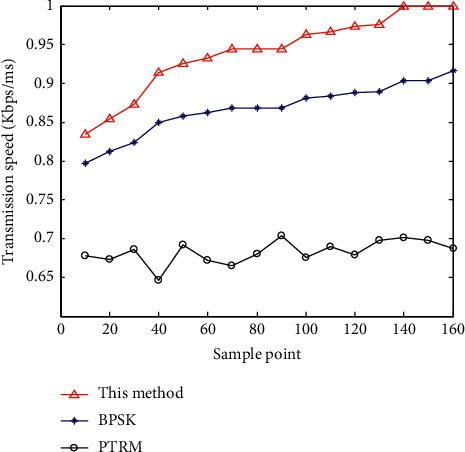
Comparison of data transmission rate.

According to analysis of Figure 6, the method proposed in this paper has good clustering and strong segmentation detection ability. On this basis, the data transmission is realized, and the data transmission rate and bit error rate are tested. Data transfer rate is one of the important technical indicators to describe the data transmission system. It refers to the speed of information transmission on the communication line and the number of bits transmitted in unit time (usually one second). Ser (symbol error rate) is an index to measure the accuracy of data transmission within a specified time. Ser = error code in transmission/total number of transmitted codes ∗ 100% [1]. If there is bit error, there is bit error rate. In addition, the bit error rate is also defined as the frequency used to measure the occurrence of errors. The research of bit error rate under specific conditions is of great significance to enhance the performance of wireless communication system and improve the quality of data transmission. The comparison results are shown in Figures 7 and 8. Through the analysis of the results in Figures 7 and 8, the bit error rate of the method proposed in this paper is maintained at about 0.05, the data transmission rate is above 0.8, and the data transmission rate continues to improve, most of which remain above 0.95. The bit error rate of BPSK and PTRM methods is above 0.1, and the data transmission rate of PTRM method is below 0.7. The results show that this method has strong anti-interference ability for the data transmission of vehicle-road cooperative communication, with low communication bit error rate, small end-to-end delay, high data transmission rate, high stability, and high accuracy.

## 5. Conclusion

The data transmission control model for vehicle-road cooperative communication is designed, which is used to improve the stability of fast data transmission of vehicle-road cooperative communication based on channel equalization adjustment. Accordingly, a fast data transmission method of vehicle-road cooperative communication based on the clustering algorithm is proposed in this paper. The single copy routing algorithm SimBet is used to reorganize the data transmission channel model for vehicle-road cooperative communication, and the data transmission channel model is thus constructed. The matched filter detection algorithm is used to realize the anti-interference design of signal transmission. According to the symbol characteristic distribution of the vehicle-road cooperative communication channel, the band interval equalization method is used to realize the segmented equalization control of the vehicle-road cooperative communication data transmission, and the channel equalization model is designed. The K-means clustering algorithm is used to carry out the coordinated adjustment to data packets during the rapid data transmission of the vehicle-road cooperative communication. According to the attenuation of communication network data transmission bandwidth, the optimal distribution interval sampling of vehicle-road cooperative communication data time series is carried out so as to improve the stability of data transmission. The test shows that the method proposed in this paper has a low bit error rate, with bit error rate kept at about 0.05, and has high data transmission rate and good stability [13].

## Data Availability

The data used to support the findings of this study are available from the corresponding author upon request.
